# Extracellular vesicles activated cancer-associated fibroblasts promote lung cancer metastasis through mitophagy and mtDNA transfer

**DOI:** 10.1186/s13046-024-03077-w

**Published:** 2024-06-03

**Authors:** Zhuan Zhou, Chunhui Qu, Peijun Zhou, Qin Zhou, Dan Li, Xia Wu, Lifang Yang

**Affiliations:** 1 Department of Oncology, Key Laboratory of Carcinogenesis and Cancer Invasion of Ministry of Education, National Clinical Research Center for Geriatric Disorders, Xiangya Hospital, Central South University, Changsha, 410078 China; 2 Cancer Research Institute, School of Basic Medicine Science, Central South University, Xiangya Road 110, Changsha, 410078 China; 3 Department of Laboratory Medicine, The Affiliated Changsha Hospital of Xiangya School of Medicine, Central South University, Changsha, 410078 China; 4 Department of Life Science, College of Biology, Hunan University, Changsha, 410012 China; 5 Department of Pathology, The Second Xiangya Hospital, Central South University, Renmin Middle Road 139, Changsha, 410011 China

**Keywords:** Extracellular vesicles, Cancer-associated fibroblasts, Mitophagy, mtDNA, Lung cancer, Metastasis

## Abstract

**Background:**

Studies have shown that oxidative stress and its resistance plays important roles in the process of tumor metastasis, and mitochondrial dysfunction caused by mitochondrial DNA (mtDNA) damage is an important molecular event in oxidative stress. In lung cancer, the normal fibroblasts (NFs) are activated as cancer-associated fibroblasts (CAFs), and act in the realms of the tumor microenvironment (TME) with consequences for tumor growth and metastasis. However, its activation mechanism and whether it participates in tumor metastasis through antioxidative stress remain unclear.

**Methods:**

The role and signaling pathways of tumor cell derived extracellular vesicles (EVs) activating NFs and the characteristic of induced CAFs (iCAFs) were measured by the transmission electron microscopy, nanoparticle tracking analysis, immunofluorescence, collagen contraction assay, quantitative PCR, immunoblotting, luciferase reporter assay and mitochondrial membrane potential detection. Mitochondrial genome and single nucleotide polymorphism sequencing were used to investigate the transport of mtDNA from iCAFs to ρ^0^ cells, which were tumor cells with mitochondrial dysfunction caused by depletion of mtDNA. Further, the effects of iCAFs on mitochondrial function, growth and metastasis of tumor cells were analysed in co-culture models both in vitro and in vivo, using succinate dehydrogenase, glutathione and oxygen consumption rate measurements, CCK-8 assay, transwell assay, xenotransplantation and metastasis experiments as well as in situ hybridization and immunohistochemistry.

**Results:**

Our findings revealed that EVs derived from high-metastatic lung cancer cells packaged miR-1290 that directly targets MT1G, leading to activation of AKT signaling in NFs and inducing NFs conversion to CAFs. The iCAFs exhibit higher levels of autophagy and mitophagy and more mtDNA release, and reactive oxygen species (ROS) could further promote this process. After cocultured with the conditioned medium (CM) of iCAFs, the ρ^0^ cells may restore its mitochondrial function by acquisition of mtDNA from CAFs, and further promotes tumor metastasis.

**Conclusions:**

These results elucidate a novel mechanism that CAFs activated by tumor-derived EVs can promote metastasis by transferring mtDNA and restoring mitochondrial function of tumor cells which result in resistance of oxidative stress, and provide a new therapeutic target for lung cancer metastasis.

**Supplementary Information:**

The online version contains supplementary material available at 10.1186/s13046-024-03077-w.

## Background

Lung cancer remains the highest incidence of mortality and the second highest incidence of morbidity among all tumors [1]. In recent years, although many advances such as surgery, radiation, chemotherapy and immunotherapies have been made in the treatment of lung cancer, locally advanced or distally metastatic tumors are still major causes of poor prognosis [2]. Therefore, the elucidation of metastatic pathways and mechanisms will provide a novel target for prediction and precisely treatment of lung cancer.

Metastasis, a multistep process involving dissemination of cancer cells from a primary site to distant tissues and organs. During the initiation of metastasis, most tumor cells undergo oxidative stress when they detach from the local extracellular matrix (ECM), resulting in cell death and the inefficiency of the metastasis [3]. However, Piskounova et al. show that circulating tumor cells (CTCs) isolated from blood and from metastatic sites with increased antioxidant capacity are able to suppress tumor cell death and promote melanoma cell metastasis [4]. Laoukili et al. demonstrate that the BRAF^V600E^ oncogene increases the capacity of disseminated tumor cells (DTCs) to withstand metastasis associated oxidative stress by stimulating glutathione synthesis. This pathway promotes the formation of liver and lung metastases [5]. Thus, metastatic tumor cells not only keep reactive oxygen species (ROS) levels higher than in primary tumor cells, but also maintain an increased antioxidant capacity, as they travel through the bloodstream and initiate new metastatic lesions [6]. Therefore, understanding how oxidative stress and its resistance in tumor cells lead to tumor metastasis will provide important evidence for elucidating the mechanism of tumor metastasis.

Mitochondria are not only the center of cellular energy metabolism, but also the main organelles involved in oxidative stress within cells. ROS can reduce the antioxidant function of mitochondria, lead to disorder of free radical metabolism, and trigger cell apoptosis by inducing mitochondrial DNA (mtDNA) damage and mediating downstream signaling [7]. In recent years, research have found that mtDNA damage is a key molecular event in oxidative stress and lead to mitochondrial dysfunction during cancer metastasis [8]. In microsatellite stabilized colorectal cancer, increasing the copy number of mtDNA can promote tumor metastasis by elevated mitochondrial oxidative phosphorylation [9]. These studies demonstrate that maintaining the integrity of mtDNA and normal mitochondrial function is essential for tumor cells during the process of metastasis [10].

Tumor metastasis is closely related to the tumor microenvironment (TME), and the cancer-associated fibroblasts (CAFs) are one of the most abundant stromal components in the TME and it critically involved in cancer progression and metastasis [11–13]. Research shows that in many types of tumors, such as breast cancer, prostate cancer, head and neck cancer and lymphoma, the ROS produced by oxidative stress in tumor cells can cause CAFs metabolic reprogramming, increase autophagy, and produce nutrients such as lactate, ketone bodies, fatty acids, glutamine, and other amino acids to support tumor cell growth and metastasis [12, 13]. Our previous studies had shown that the oncogenic protein LMP1 loaded on extracellular vesicles (EVs) secreted by nasopharyngeal carcinoma cells promotes the activation of CAFs, enhances the autophagy level of CAFs, and couples the produced metabolites with tumor cell metabolism, thereby promoting tumor metastasis and antiradiation [14]. This CAFs metabolic reprogramming phenomenon known as the reverse Warburg effect and it can be reversed after antioxidant treatment [15, 16]. Therefore, it is meaningful to study the oxidative stress resistance during tumor cell metastasis from the perspective of tumor microenvironment, especially the activation and function of CAFs.

In present study, the critical miR-1290 packaged by EVs was identified and its role of promoting normal fibroblasts (NFs) conversion to CAFs in lung cancer was demonstrated. Moreover, we found that cancer cells with damaged mitochondria could acquire mtDNA, which released by CAFs through mitophagy, resulting in recovery of mitochondria function, thereby resisting oxidative stress and promoting tumor metastasis.

## Materials and methods

### Cell culture and reagents

95C and 95D cells were obtained from the Shanghai Institute of Cell Biology (Shanghai, China). The paired cells are human giant cell lung carcinoma cell lines, and have the same genetic background and varied metastatic capacity. 95D cells had the higher metastatic capacity [17]. The cells were cultured in DMEM (Gibco BRL, Gaithersburg, MD, USA) supplemented with 10% fetal bovine serum (FBS, Hyclone, Logan, UT, USA). A549 (CRM-CCL-185) and H1299 (CRL-5803) cells were obtained from the American Type Culture Collection (ATCC, Manassas, VA, USA). A549 was cultured in DMEM/F12 (Gibco BRL) supplemented with 10% FBS, and H1299 was cultured in DMEM supplemented with 10% FBS at 37 °C in a 5% CO_2_ incubator.

Primary NFs and CAFs were cultured in our laboratory [18]. For the preparation of ρ^0^ cells which was depleted of mitochondrial DNA, parental tumor cell lines A549 and H1299 cells were cultured for 30 or 60 days in low-dose Ethidium bromide (EtBr, 50 ng/mL) supplemented with 100 μg/mL pyruvate and 50 μg/mL uridine. After 60 days of culture, ρ^0^ cells were transfer to medium lacking EtBr [19]. EtBr (E1510), pyruvate (P5280), uridine (U3003), DNaseI (D7291), Carbonyl cyanide 3-chlorophenylhydrazone (CCCP, C2759) and hydrogen peroxide (H_2_O_2_, 516813) were purchased from Merck Millipore (Billerica, MA, USA).

### EVs isolation

EVs-depleted FBS was purchased from SBI (Palo Alto, CA, USA). Cells were cultured in DMEM with 10% EVs-depleted FBS at 37 °C for 48 h, and their culture media were collected for EVs isolation. The isolation of EVs was performed using ExoQuick-TC™ kit (EXOTC10A-1, SBI). BCA Assay reagent (23225, Pierce™ Thermo Fisher, Waltham, MA, USA) was used to measure the concentration of isolated EVs. The purified EVs resuspended in PBS were stored at − 80 °C.

### Transmission electron microscopy (TEM)

The morphology of EVs was evaluated using a TEM (H-600, Hitachi, Tokyo, Japan) at a voltage of 75 kV. EVs sample was diluted with PBS and dropped on a carbon-coated copper grid. To remove unattached EVs, the grid was washed with ddH_2_O. After staining with a 2% phosphotungstic acid, the grids were air-dried and subjected to TEM assay.

### Nanoparticle tracking analysis (NTA)

NTA of EVs samples was carried out using a Zeta View PMX 120 (Particle Metrix, Meerbusch, Germany) as previously described [14]. Briefly, EVs were diluted (v/v, 1:4000) with ddH_2_O, and their size distributions and particle concentrations were analyzed using the parameters (min area 5, max area 1000, min brightness 20, and camera 0.713 μm/px) at 25 °C.

### Intracellular uptake of EVs

PKH67 (MINI67, Sigma-Aldrich, St. Louis MO, USA) fluorescent staining was performed to label EVs. The PKH67-labeled EVs were cocultured with NFs for 24 h in FBS-free DMEM medium at 37 °C. The cells were fixed with 4% paraformaldehyde solution for 10 min, and then the cells were incubated with phalloidin (C2207S, Beyotime, Shanghai, China) for 30 min. DAPI staining solution (E607303, BBI Life Sciences, Shanghai, China) was used to stain the cell nucleus. The stained cells were observed under a laser scanning confocal microscopy (LSM900, Zeiss, Oberkochen, Germany).

### Transfection

The sequences of the mimics (miR-1290, miR-652, miR-222), inhibitors (miR-1290), and negative control were all synthesized from Ribobio Company (Guangzhou, China). The pDONR233-MT1G plasmid and empty plasmid were purchased from Youbio (G152804, Hunan, China). For the transient transfection of plasmid and miRNA-mimic/inhibitor, Lipofectamine^3000^ reagent (L3000015, Invitrogen, Carlsbad, CA, USA) was used to according to the manufacturer's instructions.

### Quantitative PCR (qPCR)

Trizol reagent was purchased from Thermo Fisher Scientific (15596026) to extract total RNA from cells and EVs according to the manufacturer’s instructions, and cel-miR-39 (Ribobio) was added into each EVs sample at a final concentration of 10 pmol/μL acting as external reference. Reverse transcription was performed using Mir-X™ miRNA First-Strand Synthesis Kit for miRNAs (638315, Clontech, Mountain View, CA, USA) or RevertAid First Strand cDNA Synthesis Kit (K1621, Thermo Fisher Scientific) for general genes. For mitochondrial DNA isolation, DNA was extracted from cells or cell-free supernatants with QIAamp DNA Blood Mini Kit (Qiagen, Duesseldorf, Germany). DNA was extracted from formaldehyde-fixed mouse tissue using the TIANamp FFPE DNA Kit (TIANGEN, DP331, Beijing, China) according to the manufacturer’s instructions. qPCR was conducted using SYBR Green (4309155, Life Technologies Corporation, Gaithersburg, MD, USA) and performed on ABI 7500 PCR detection system (Foster City, CA, USA). The sequences of all indicated primers were listed in Table S1-S2. The relative expression levels of mRNAs, miRNA and mtDNA were calculated with 2^–ΔΔCt^ method. Actin was selected as the housekeeping gene. U6 and miR-39 were selected as the housekeeping gene of miRNA for cells and EVs, respectively.

### Immunoblotting

The cells were lysed in IP buffer (87787, Thermo Fisher Scientific) containing inhibitors cocktail (4693116001, Roche, Basel, Switzerland). The protein concentration was measured by BCA Assay reagent (23225, Pierce Chemical). The protein was separated by SDS-PAGE and then transferred to PVDF membrane (Merck Millipore). The membrane was incubated with first antibody at 4 °C overnight, and then incubated with the second antibody 1 h. The bands were observed with an enhanced chemiluminescence detection kit (36208-A, Yeasen, Shanghai, China) and analyzed by ChemiDoc XRS system (Bio-Rad, Hercules, CA, USA).

The antibodies were used as follows, α-SMA (14395–1-AP), Vimentin (10366–1-AP), PINK1 (23274–1-AP), AKT (60203–2-IG), p-AKT (66444–1-IG), Calnexin (10427–2-AP), E-cadherin (20874–1-AP) and Snail (13099–1-AP) were purchased from Proteintech (Wuhan, China). Actin (AC026), GAPDH (AC002), and FAP (A6349) were purchased from Abclonal (Wuhan, China). MT1G (CSB-PA17384A0Rb) was purchased from Cusabio (Wuhan, China). LC3 (L7543) was purchased from Sigma-Aldrich. BNIP3 (Ab10433), CD63 (Ab134045) and TSG101 (Ab125011) were purchased from Abcam (Cambridge, MA, USA).

### Immunofluorescence (IF)

The cells were fixed with 4% paraformaldehyde solution for 10 min, and then blocked in 5% donkey serum in PBS for 1 h and incubated with the primary antibody (α-SMA, 55135–1-AP, Proteintech) at 4˚C overnight. The cells were incubated with fluorochrome-conjugated secondary antibody (Anti-Rabbit, SAB4600234, Sigma-Aldrich) for 45 min. DAPI staining solution (E607303, BBI Life Sciences) was used to stain the cell nucleus. For fluorescence analysis, cell samples were visualized on a laser scanning confocal microscopy (LSM900, Zeiss).

### Collagen contraction assay

A total of 2 × 10^5^ NFs were suspended in 100 μL DMEM. Then the cell suspension was mixed with 400 μL of cold collagen gel working solution (Cell Contraction Assay, CBA-201, Cell Biolabs, San Diego, CA, USA), added to one well of 24-well plates and allowed to solidify for 60 min at 37 °C. After collagen polymerization, 1.0 mL of culture medium containing EVs is added atop each collagen gel lattice. After incubation two days with medium, gently release collagen gels from the sides of the culture dishes with a sterile spatula, the gels were photographed by digital camera.

### Luciferase reporter assay

The 3′ UTR segments of the MT1G genes were amplified by PCR and inserted into the vector pmiR-RB-REPORT™ (Ribobio). Co-transfections of MT1G 3′ UTR plasmids with miR-1290 mimic into the cells were accomplished by using Lipofectamine^3000^ (L3000075, Invitrogen). Luciferase activity was measured 48 h after transfection by the Dual-Luciferase Reporter Assay System (E1910, Promega, Madison, WI, USA).

### Mitochondrial membrane potential (MMP) detection

After 2 × 10^4^ cells were treated in a 6-well plate, JC-1 dye (J6004L, Uelandy, Suzhou, China) was added for 20 min at 37 °C. MMP was detected and analyzed using relative fluorescence ratio staining. The red to green fluorescence ratio was lower in damaged mitochondria cells than in normal cells. The fluorescence intensity was measured by BioTek Cytaiton1 (Hercules, CA, USA).

### Succinate dehydrogenase (SDH) and Glutathione (GSH) assay

SDH activity was measured using Succinate Dehydrogenase Activity Assay Kit (D799375, Sangon, Shanghai, China). GSH concentration was measured using GSH Content Assay Kit (D799614, Sangon). All assays were performed following the manufacturer’s instruction. SDH activity was measured by absorbance at 600 nm and GSH was detected by absorbance at 412 nm using BioTek Cytaiton1 (Hercules).

### Oxygen consumption rate (OCR) assay

Cells were seeded in XFe 96-well microplates (6000 cells/well) (Agilent Technologies, Sana Clara, USA), followed by indicated stimulation for a further 48 h. Cells were washed and incubated in base medium (Agilent Technologies) at 37 °C for 1 h. OCR was measured in real-time with Mito Stress Test Kit (103015–100, Agilent Technologies) using the Seahorse XFe96 Analyser (Agilent Technologies) following manufacturer’s instructions.

### Reactive oxygen species (ROS) assay

Cells after treated were collected, washed with PBS twice, and incubated with DCFH-DA (S0033S, Beyotime) at a final concentration of 10 μM in FBS-free DMEM for 15 min at 37 °C in the dark, and then washed three times with FBS-free DMEM. The cells were collected, and ROS levels were analyzed using flow cytometer (FlowSight, Millipore, Billerica, MA, USA).

### Mitochondrial genome sequencing

Total DNA was extracted from cells with QIAamp DNA Blood Mini Kit (Qiagen) according to the manufacturer’s recommendations. The DNA samples were quantified with NanoDrop 2000 (Thermo Fisher Scientific) and ascertained with electrophoresis. The mitochondria genome was sequenced by Genesky Biotechnologies (Shanghai, China).

### Single nucleotide polymorphism (SNP) sequencing

Using H1299ρ^0^ cells as control, NFs treated with 95D-EVs and H1299ρ^0^ cells cocultured with conditional medium (CM) from NFs treated with 95D-EVs, DNA of these three groups was extracted with QIAamp DNA Blood Mini Kit (Qiagen) and sequenced by Sangon Biotech (Shanghai, China) to analyze the variation of single nucleotide in the genome.

### Mitochondrial function

Cells were incubated with Mito-Red (M7512, Invitrogen) at a final concentration of 25 nM for 15 min at 37 °C, and subsequently stained with DAPI (E607303, BBI). Next, images were captured and analyzed using a laser scanning confocal microscopy (LSM900, Zeiss).

### CCK8 assay

According to the manufacturer's instructions, the cell viability was measured through the CCK8 kit (C0005, Targetmol, Boston, MA). The cells of the control group or the treatment group were seeded in a 96-well plate for an appropriate time, the viability was measured OD450 after 2 h incubation with determination solution using Microplate Reader (BioTek ELx800, Winooski, VT).

### Transwell assay

1 × 10^5^ lung cancer cells were suspended in serum-free medium and seeded into the transwell chambers with inserts of 8-μm pore size (353097, Falcon, NY, USA). The medium with 10% FBS was placed into the bottom chamber (353,047, Falcon). After 24 h, the cells that had migrated through the membrane and stuck to the lower surface of the membrane were stained with crystal violet solution (V5265, Sigma-Aldrich) and were measured by light microscope (AMEX-1200, AMG).

### Animal experiment

H1299 (2 × 10^6^), H1299ρ^0^ cells (2 × 10^6^), the mixture of 2 × 10^6^ H1299ρ^0^ cells with 5 × 10^5^ NFs or 5 × 10^5^ induced CAFs (iCAFs, that originated from NFs treatment with 95D-EVs) cells (4:1) was resuspended in 100 μL PBS and injected subcutaneously into 5-week-old female BALB/C nude mice as previously described [18]. Tumor formation was examined every 3 days. The tumor volume was calculated as volume (mm^3^) = d^2^ × D/2, where d and D were the shortest and the longest diameters, respectively.

To examine the roles of EVs in metastasis, 5 × 10^5^ H1299 cells were intravenously injected into 5-week-old female BALB/c nude mice through the tail vein. Subsequently, mice were randomly divided into groups and intravenously injected with equal numbers of EVs from different tumor cells twice a week for 1 month [20].

For lung metastasis experiments, the nude mice were randomly divided into four groups, H1299 (5 × 10^5^), H1299ρ^0^ cells (5 × 10^5^), the mixture of 5 × 10^5^ H1299ρ^0^ cells with 5 × 10^5^ NFs or 5 × 10^5^ iCAFs cells (1:1) were injected 5-week-old female BALB/c nude mice via the tail vein, respectively [21]. The nude mice were sacrificed by cervical dislocation at the indicated timepoint, and lung tissue was removed, imaged and the number of nodules on the surface of the lung was recorded to assess tumor metastasis. Lung tissues were then fixed with 4% paraformaldehyde for hematoxylin–eosin (H&E).

### In situ hybridization (ISH) and immunohistochemistry (IHC)

Tissue sections of 41 clinical lung cancer pathological sections from the Department of Pathology of the second Xiangya Hospital (2019–2021) were collected (Table S3). For ISH, the miR-1290 miRCURY LNA detection probe (Exiqon, Denmark) was used by the manufacturer’s protocol. IHC was performed using universal two-step detection kit (PV-9000, ZSGB-Bio, Beijing). The sections were stained with DAB (ZLI-9017, ZSGB-Bio, Beijing) for 1–3 min, and lightly counterstained with Mayer hematoxylin (ZLI-9610, ZSGB-Bio). The expression of each protein was semi-quantitatively evaluated using the method previously described [14]. TOM20 (11802–1-AP, Proteintech) was used in this experiment.

### Statistical analysis

Statistical analyses were performed using GraphPad Prism 8 software. Quantitative values of all experiments were expressed as the mean ± SD. Differences among/between sample groups were analyzed by one-way ANOVA or the independent samples T test. Pearson’s correlation coefficient was used to measure the relationship between miR-1290, α-SMA and BNIP3. *P* < 0.05 was considered to be statistically significant.

## Results

### High metastatic potential lung cancer cells derived EVs packaged miR-1290 activates NFs to CAFs

**Fig. 1:**
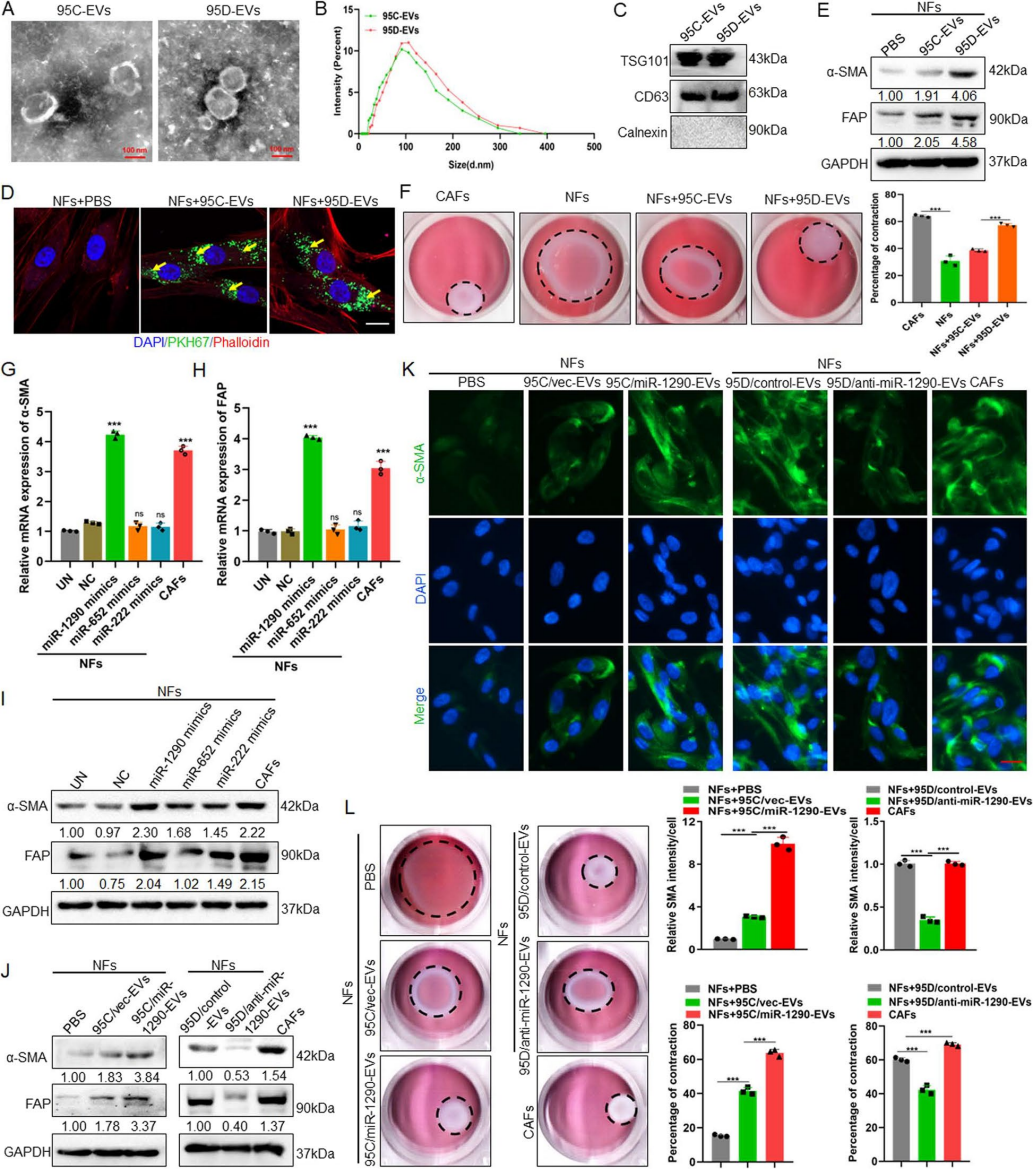
95D-derived EVs packaged miR-1290 activated NFs to CAFs.** A** The representative images of EVs derived from 95C and 95D were analyzed by TEM. Scale bar, 100 nm. **B** EVs were detected by NTA analysis. **C** Immunoblotting analyses for EVs markers CD63, TSG101 and negative control Calnexin. **D** NFs were pre-treated with 95C or 95D derived EVs for 24 h. Confocal imaging showed the delivery of PKH67-labeled EVs (green) to Phalloidin labelled NFs (red) and the localization of DAPI-stained nuclei (blue). Yellow arrows represented delivered EVs. Scale bar, 20 μm. **E** After NFs were incubated with 95C or 95D derived EVs for 48 h, the protein levels of α-SMA and FAP were analyzed by immunoblotting. **F** Representative images of the contraction ability were analyzed by collagen contraction assay. Isolated primary CAFs were used as the positive control group. **G-H** After NFs transfected with indicated mimics, the mRNA levels of α-SMA and FAP were analyzed by qPCR. Negative control (NC), Untreated (UN). **I** The protein levels of α-SMA and FAP were detected by immunoblotting. **J** Immunoblotting analysis of α-SMA and FAP in NFs treated with blank control (PBS) or EVs derived from 95C/vec and 95C/miR-1290 or 95D/control and 95D/anti-miR-1290. **K** Representative images and quantification analysis of IF of α-SMA in NFs treated as indicated. Scale bar, 20 μm. **L** Collagen contraction assay was assessed for the contraction ability of NFs treated as indicated. Data were shown as the mean ± SD of at least three independent experiments. ***P < 0.001, ns: not significant

CAFs have been demonstrated to participate in the tumor metastasis progression. Therefore, it was meaningful to further understand the activation of CAFs caused by tumor cells derived EVs. The primary isolated NFs and CAFs from lung tissue were constructed in our previous study [18]. Both NFs and CAFs expressed vimentin, while NFs had decreased expression of the CAFs markers a-SMA and FAP proteins (Fig. S1). To determine the difference of EVs derived from low metastatic potential 95C and high metastatic potential 95D cells in CAFs activation, firstly, EVs derived from cells were isolated and purified, the result showed that both isolated EVs exhibited typical lipid bilayer membrane encapsulation and cup-shaped morphology by TEM analysis (Fig. 1A). The size distribution of EVs ranged from 30 to 300 nm, and the peak was approximately 110 nm by NTA (Fig. 1B). Additionally, immunoblotting revealed that the EVs were positive for EVs markers CD63 and TSG101 (Fig. 1C). To identify the internalization of EVs by NFs, the tumor-cell derived EVs and NFs were labeled with PKH67 (green) or Phalloidin (red), respectively. As shown in Fig. 1D, EVs were internalized and accumulated around the nuclei of NFs after incubation, suggesting that EVs were effectively delivered to NFs. Meanwhile, the expression of α-SMA and FAP in NFs were significantly higher in the 95D-derived EVs treatment groups than in the 95C-derived EVs groups (Fig. 1E, Fig. S2). These data indicated that NFs were activated by 95D-derived EVs. It is known that activated fibroblasts have enhanced matrix adhesions, resulting in increased ability of collagen gel contraction [14]. Further data showed that the contraction abilities of NFs were markedly enhanced after treatment with 95D-derived EVs in comparison with 95C-derived EVs (*P* < 0.001) (Fig. 1F). These results suggested that 95D-derived EVs could effectively activate NFs to CAF-like cells.

miRNAs encapsulated in EVs are abundant and have an important role in cell–cell communication. Previously, we conducted microarrays to generate miRNAs profiles between the 95D and 95C cells and found 16 miRNAs with significantly different expression levels [17]. To identify the specific miRNAs involved NFs activation, qPCR was used to examine the expression of these 16 miRNAs in the EVs, the results showed that miR-222, miR-487b, miR-652, miR-487a, miR-1290 and miR-663 were highly expressed in 95D cell derived EVs (Fig. S3A). Meanwhile, these 6 miRNAs were detected in NFs and CAFs cells, and the results showed that miR-222, miR-652 and miR-1290 were highly expressed in CAFs, suggesting that these 3 miRNAs might be associated with the activation of NFs (Fig. S3B). To confirm this, the expression of α-SMA and FAP at both mRNA and protein levels after transfecting miR-222, miR-652, miR-1290-mimics in NFs cells were detected, the results suggested that the miR-1290-mimics could promote the expression of α-SMA and FAP (Fig. 1G, H, I). Further, the stable cell lines which overexpressing miR-1290 in 95C cell or knockdown miR-1290 in 95D cells were established (Fig. S4). After NFs cocultured with EVs, the data demonstrate that 95C/miR-1290-derived EVs could increase the expression of α-SMA and FAP, while EVs secreted from 95D/anti-miR-1290 could decrease two protein expression (Fig. 1J). These results were further confirmed by IF assays for α-SMA (Fig. 1K). Furthermore, collagen contraction assays also revealed that EVs packaged miR-1290 enhanced the collagen contraction ability of NFs (Fig. 1L). These results suggest that EVs packaged miR-1290 could activate NFs to CAFs.

#### *EVs packaged miR-1290 activates NFs to CAFs *via* MT1G/AKT *and promotes lung cancer metastasis

**Fig. 2:**
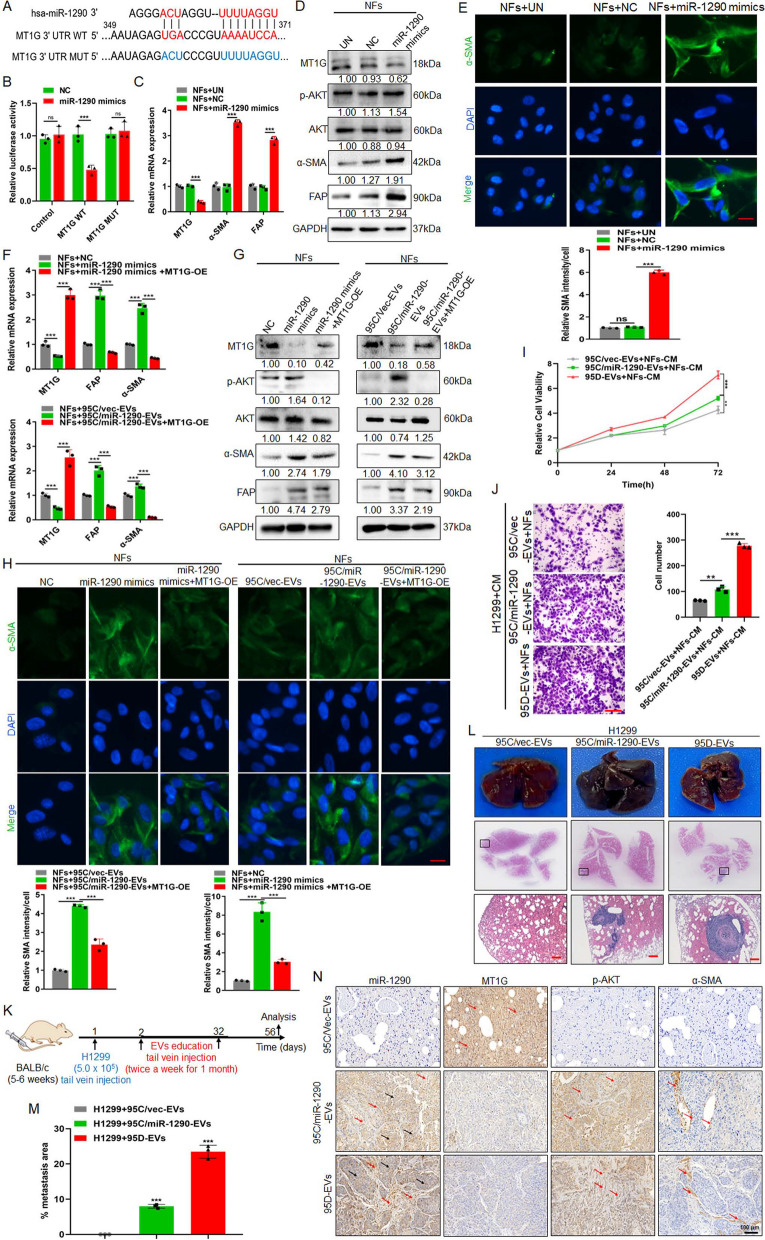
EVs packaged miR-1290 activates NFs to CAFs via MT1G/AKT and promotes lung cancer metastasis. **A** The binding sites and corresponding mutation sites between miR-1290 and the target gene MT1G. **B** Luciferase reporter assay in 293 T cells cotransfected with the wild or mutant-type MTIG 3' UTR and miR-1290 mimics. Untreated (UN). Blank Vector (Control). **C** qPCR analysis of MT1G, α-SMA and FAP expression in NFs treated with miR-1290 mimics. Negative control (NC). **D** Immunoblotting analysis of MT1G, p-AKT, AKT, α-SMA and FAP in NFs transfected with miR-1290 mimics. **E** IF analysis and quantification data of α-SMA in NFs treated as indicated. Scale bar, 20 μm. **F–H** pDONR233-MT1G plasmid was transfected in NFs treated with miR-1290 mimics and 95C/miR-1290-EVs, respectively, **F** qPCR assays for MT1G, α-SMA and FAP, **G** Immunoblotting analysis of MT1G, p-AKT, AKT, α-SMA and FAP, **H** IF analysis for α-SMA. Scale bar, 20 μm. **I-J** H1299 was cocultured with conditional medium (CM) from different groups indicated, **I** CCK8 assay for cell proliferation, and **J** Transwell assays for the cell migration analysis. Scale bar, 100 μm. **K** Schematic representation of the establishment process of the EVs-educated tumor metastasis model. **L** The representative tissue images and HE images of lung foci. Scale bar, 200 μm. **M** Quantitative metastatic area in lung metastatic foci. **N** ISH images of miR-1290 and IHC images of MT1G, p-AKT and α-SMA in lung foci of different experimental groups. Red arrow: positive signal in the stroma; Black arrow: positive signal in the tumor. Data were shown as the mean ± SD of at least three independent experiments.**P < 0.01,  ***P < 0.001, ns: not significant

A study in lung cancer showed that Metallothionein 1G (MT1G), as a target for miR-1290, have critical roles in regulating tumor growth and metastasis [22]. Consistent with previous study, we confirmed that miR-1290 could mediate MT1G expression through luciferase reporter assay (Fig. 2A, B). Further, qPCR results showed that MT1G expression could be downregulated, and α-SMA and FAP expression were upregulated in NFs treated with miR-1290 mimics compared to controls (Fig. 2C). The previous study had reported that MT1G could inhibit AKT signaling [23], and AKT signaling pathway was responsible for NFs activation [24]. As shown in Fig. 2D, miR-1290 mimics could suppress the expression of MT1G, meanwhile activating the AKT and upregulating the expression of α- SMA and FAP in NFs. IF results also showed that overexpressed miR-1290 in NFs could promoted α-SMA expression (Fig. 2E). Furthermore, to explore the importance of MT1G/AKT in the activation of CAFs, functional rescue experiments showed that restoration the expression of MT1G in miR-1290 mimics-treated NFs could suppress the expression of the p-AKT, α-SMA and FAP. In addition, 95C/miR-1290-EVs increased the protein level of p-AKT, α-SMA and FAP, while restoration of MT1G expression abrogated these effects (Fig. 2F, G). Similar results were observed in IF staining experiments (Fig. 2H). Moreover, over-expressed MT1G in CAFs decreased the protein level of p-AKT, α-SMA, FAP and the intensity of α-SMA (Fig. S5A, B, C).

To explore the function of EVs-packaged miR-1290 in tumor metastasis, NFs cells were firstly treated with EVs derived from 95C/vec, 95C/miR-1290 and 95D cells, then co-culture the collected CM with H1299 cells. CCK8 and Transwell assays revealed that 95C/miR-1290 and 95D-derived EVs could notably promote the proliferation and migration of cancer cells in vitro (Fig. 2I, J). For in vivo lung metastatic mouse model, H1299 was injected in mice tail vein firstly, then EVs derived from 95C/vec, 95C/miR-1290, and 95D were injected respectively, lung metastasis foci were observed after 8 weeks (Fig. 2K). The results showed that, compared with 95C/vec-EVs group, 95C/miR-1290 as well as 95D-derived EVs promoted the formation of lung metastases more strongly (Fig. 2L, M). In addition, compared with that in 95C/vec-EVs-treated group, a dramatically upregulated expression of miR-1290, p-AKT and α-SMA was observed in lung from mice implanted 95C/miR-1290 derived EVs, while MT1G was decreased (Fig. 2N). Collectively, these results suggest that EVs-packaged miR-1290 promoted the activation of NFs to CAFs by targeting MT1G/AKT, and accelerated the metastatic homing of lung cancer cells.

#### ROS enhances mitophagy and increases release of mtDNA in iCAFs

**Fig. 3:**
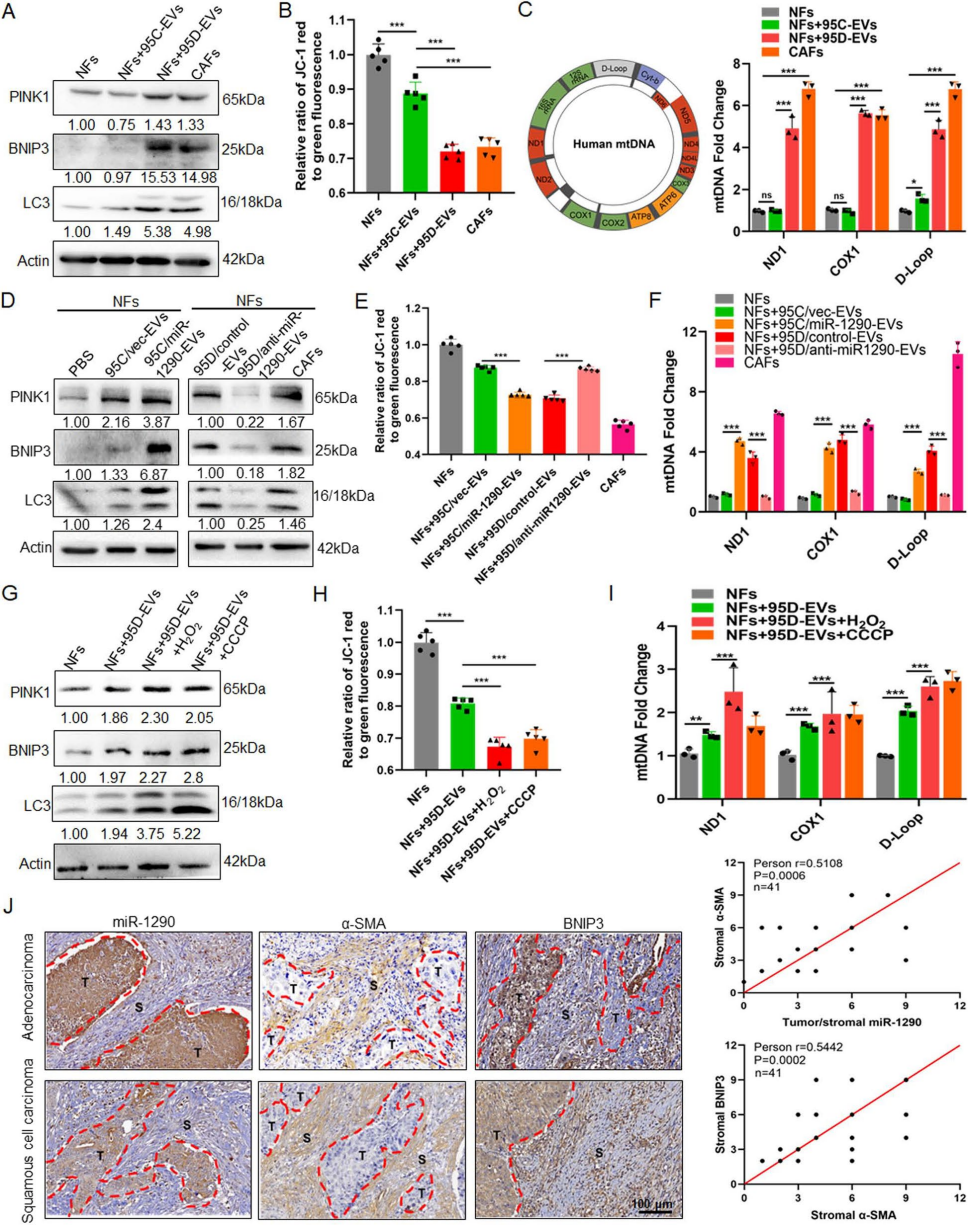
ROS enhances mitophagy and increases release of mtDNA in iCAFs. **A-C** NFs were treated with EVs derived from 95C or 95D for 48 h. Isolated NFs and CAFs were used as blank control and positive control, respectively. **A** Immunoblotting analysis of PINK1, BNIP3 and LC3 in the indicated groups. **B** The fluorescence intensity reflecting the MMP in the different groups. **C** qPCR analyses for ND1, COX1, D-Loop in the supernatants of indicated cells. **D** Immunoblotting analysis of PINK1, BNIP3 and LC3 in NFs treated with blank control (PBS) or EVs derived from 95C/vec and 95C/miR-1290 or 95D/control and 95D/anti-miR-1290. **E** The fluorescence intensity reflecting the MMP in NFs treated as indicated. **F** qPCR analyses for ND1, COX1, D-Loop in the supernatants of indicated cell. **G-I** NFs were treated with 95D-derived EVs for 48 h, and then exposed to H_2_O_2_ (0.25 mM) or CCCP (10 μM), respectively. **G** Immunoblotting analysis of PINK1, BNIP3 and LC3 in the indicated groups. **H** The fluorescence intensity reflecting the MMP in the different groups. **I** qPCR analyses for ND1, COX1, D-Loop in the supernatants of indicated cells. **J** ISH images of miR-1290 and IHC images of α-SMA and BNIP3 in lung cancer tissue sections. Stromal and tumor are separated by dashed lines (left). The correlation analysis (right) between miR-1290 and α-SMA (upper), and between α-SMA and BNIP3 (lower). Data were shown as the mean ± SD of at least three independent experiments. *P < 0.05, **P < 0.01, ***P < 0.001,  ns: not significant

To investigate the autophagic status of iCAFs that originated from NFs treatment with 95D-EVs, autophagy-related protein LC3 and mitophagy-related proteins BNIP3 and PINK1 were detected. The result showed that the expression of LC3, BNIP3 and PINK1 was overexpressed in iCAFs (Fig. 3A). MMP was regard as a key indicator of mitochondrial function [25]. Here, the loss of MMP levels was observed in iCAFs compared with NFs cocultured with 95C-EVs, indicating that iCAFs had a weaker mitochondrial function (Fig. 3B). Further, qPCR analysis for ND1, COX1 and D-Loop was used to detect the released mtDNA in cell culture medium, the results showed that more mtDNA was released in iCAFs group (Fig. 3C). Further, treatment with 95C/miR-1290-EVs dramatically promoted mitophagy status, while treatment with 95D/anti-miR-1290-EVs significantly decreased the mitophagy of iCAFs (Fig. 3D). Consistently, the loss of MMP levels was observed in NFs treated 95C/miR-1290-EVs, and reversed expression pattern was found in NFs cocultured with 95D/anti-miR-1290-EVs (Fig. 3E). qPCR data showed that NFs treatment with 95C/miR-1290-EVs released more mtDNA, while 95D/anti-miR-1290-EVs blocked the effect of mtDNA release from iCAFs (Fig. 3F). Study had demonstrated that cells stimulated by hydrogen peroxide (H_2_O_2_) could generate high levels of ROS that can perturb the normal redox balance and shift cells into a state of oxidative stress [26]. Therefore, H_2_O_2_ was used to established a model of oxidative stress in vitro. It was shown that the mitophagy in iCAFs was enhanced in a concentration dependent manner (Fig.S6A). Meanwhile, the result showed that CCCP, a mitophagy inducer, could significantly increase mitophagy of iCAFs (Fig. S6B). Interestingly, iCAFs dramatically elevated mitophagy status compared with NFs cocultured with 95C-EVs, and H_2_O_2_ can further enhance mitophagy of iCAFs (Fig. S6C). As shown in Fig. 3G  and H, H_2_O_2_ treatment of iCAFs promoted the mitophagy and decreased the level of MMP, which is the same as the result of positive control CCCP treatment. Similarly, qPCR data showed that iCAFs released more mtDNA compared to NFs, and H_2_O_2_ treatment further promoted the release of mtDNA (Fig. 3I). In addition, primary human lung cancer tissues were examined to seek evidence for tumor derived miR-1290 activated CAFs as well as the consequent high mitophagy. miR-1290 levels of tumor and stromal was positively correlated with stromal α-SMA, whereas in the stroma, a strong correlation between α-SMA and BNIP3 (Fig. 3J). These data suggested that iCAFs exhibited higher levels of autophagy and mitophagy and more mtDNA released, and ROS could further enhance this process.

#### iCAFs transports mtDNA to tumor cells with damaged mitochondria

**Fig. 4:**
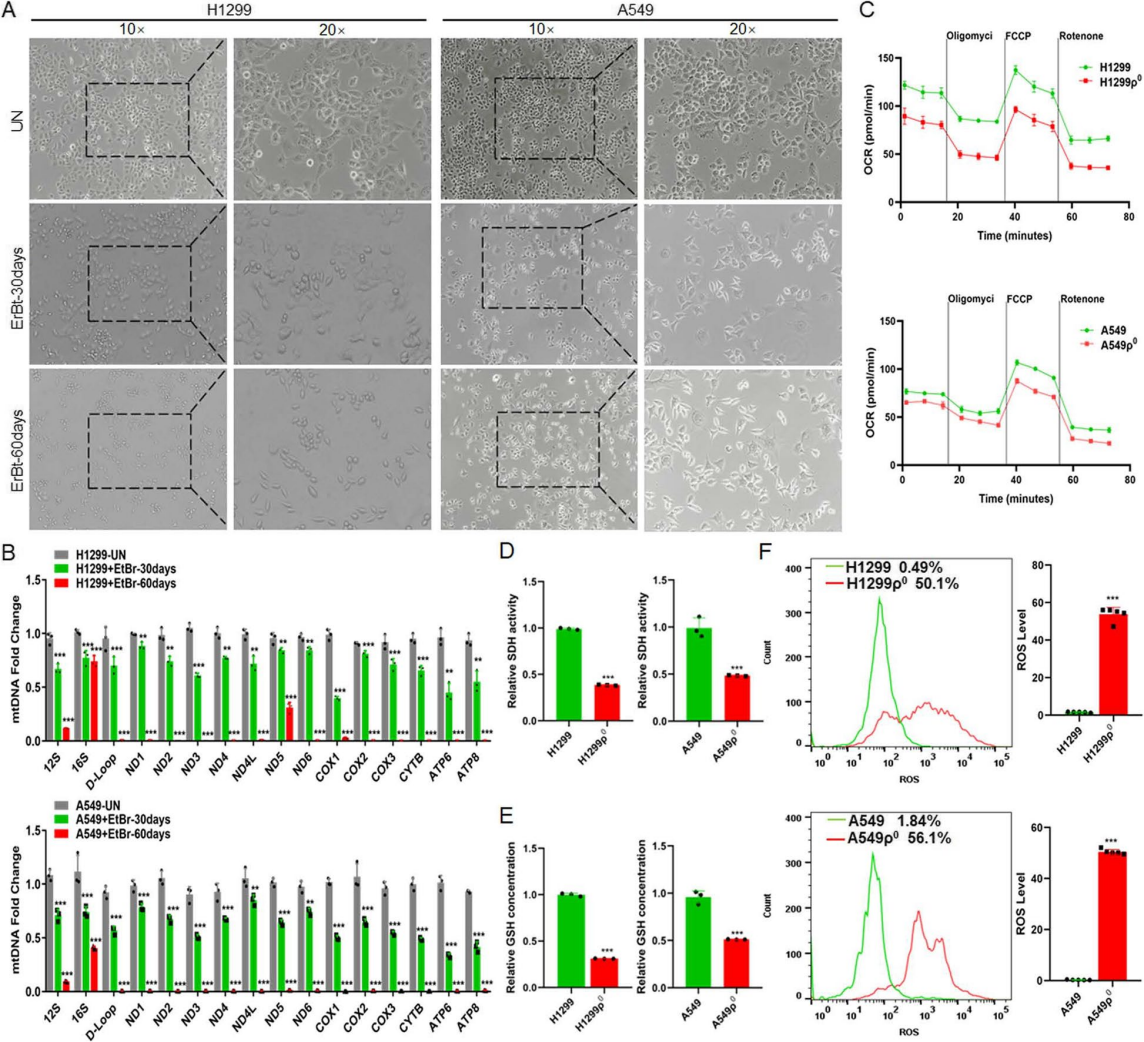
The differences between normal and mitochondria damaged lung cancer cells. **A** The representative macrographs of parental cells and cells treated with EtBr for 30 or 60 days. **B** qPCR analysis of 16 genes (12S, 16S, D-Loop, ND1, ND2, ND3, ND4, ND4L, ND5, ND6, COX1, COX2, COX3, CYTB, ATP6, ATP8) in cells**.** Untreated (UN). **C** OCR was detected by seahorse mitochondrial stress in indicated groups. ρ^0^ cells: cells were depleted of mitochondrial DNA after treated with EtBr 60 days. **D, E** The activity of SDH and the concentration of GSH were detected. **F** The level of ROS in cells was analyzed by flow cytometry. Data were shown as the mean ± SD of at least three independent experiments. **P < 0.01, ***P < 0.001

To construct tumor cells with damaged mitochondria, lung cancer cells H1299 and A549 cells were treated with low-dose EtBr in order to generating mtDNA depletion cell (H1299ρ^0^ and A549ρ^0^ cells). After 30 and 60 days of cell treatment, morphological observation showed gradually enhanced cytoplasmic shrinkage (Fig. 4A). qPCR was performed to detect the expression of mtDNA-encoded genes including 12S, 16S, D-loop, ND1, ND2, ND3, ND4, ND4L, ND5, ND6, COX1, COX2, COX3, CYTB, ATP6, ATP8. The results demonstrated that the vast majority genes were obviously decreased in H1299ρ^0^ and A549ρ^0^ cells (Fig. 4B). Then, cellular OCR and oxidative stress-related enzymes GSH and SDH was detected, the results showed that the level of the OCR, SDH activity and GSH concentration in H1299ρ^0^ and A549ρ^0^ cells were lower than that in parent tumor cells (Fig. 4C, D, E). Studies have demonstrated that the ROS levels in EtBr-treated cells were significantly higher than in the controls [27]. Our data is consistent with previous studies that cellular ROS were increased in H1299ρ^0^ and A549ρ^0^ cells (Fig. 4F). The above results showed that tumor cell with depleted mtDNA exhibit mitochondrial dysfunction.

**Fig. 5:**
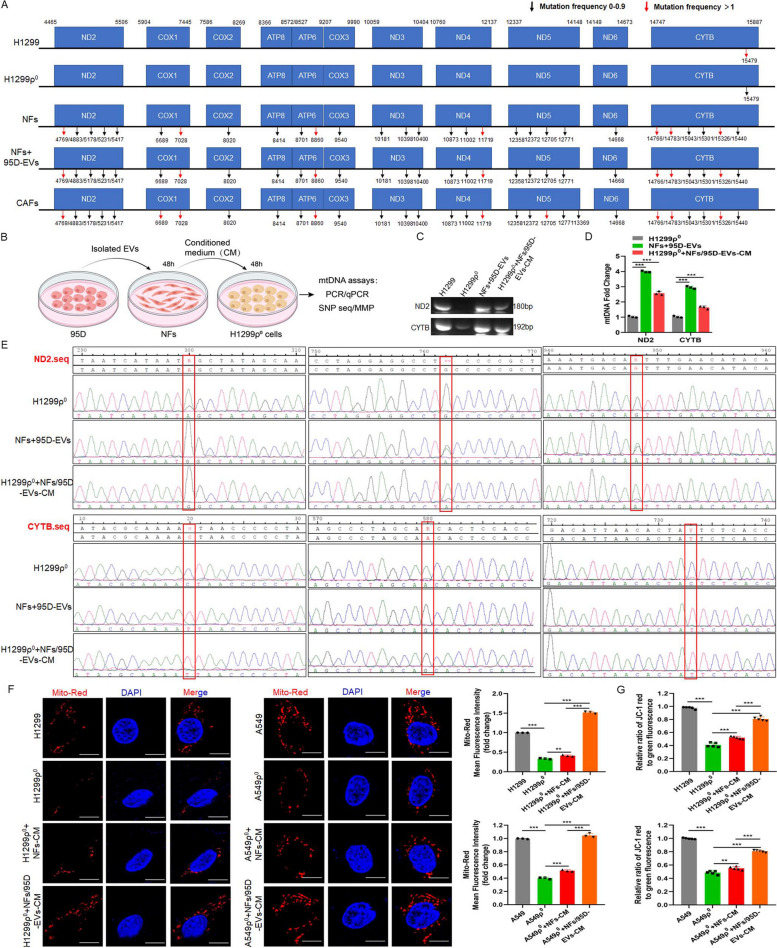
iCAFs transports mtDNA to tumor cells with mitochondria damaged.** A** Mitochondrial genome sequencing was performed in H1299, H1299ρ^0^, NFs, iCAFs (NFs treated with 95D-EVs for 48 h) and isolated primary CAFs. **B** Diagram of experimental procedure for inducing CAFs and coculturing with H1299ρ^0^ cells. **C, D** PCR and qPCR were used to analyze the ND2 and CYTB in the indicated groups. **E** SNP sequencing of the ND2 and CTYB was performed. **F** Confocal imaging showed mitochondria labeled with mito-Red and the localization of DAPI-stained nuclei (blue) in the indicated groups. Mean fluorescence intensity (Mito-Red) data was shown. Scale bar, 10 μm. **G** The fluorescence intensity reflecting the MMP in the different groups. Data were shown as the mean ± SD of at least three independent experiments. **P < 0.01, ***P < 0.001

To determine whether the iCAFs transmitted mtDNA to mitochondria damaged cells, the whole mitochondrial DNA sequencing was performed for H1299, H1299ρ^0^, NFs, iCAFs and primary CAFs. The results showed that the sequences of mtDNA in H1299ρ^0^ cells was not obvious different from that in H1299, and the mtDNA from stromal cells (NFs, iCAFs and CAFs) had different degrees of mutations compared to H1299. In addition, the mtDNA of iCAFs also showed a basically consistent mutation frequency compared with CAFs (Fig. 5A). To elucidate whether tumor cells can obtain mtDNA from CAFs, the CM from iCAFs was isolated and cocultured with H1299ρ^0^ cells (Fig. 5B), the results showed that the expression of ND2 and CYTB in tumor cells was restored (Fig. 5C, D). Further, mtDNA SNP sequencing of H1299ρ^0^, iCAFs and H1299ρ^0^ cocultured with iCAFs-CM was performed, the results displayed that compared to H1299ρ^0^, both ND2 as well as CYTB had three mutant sites in the iCAFs group, and specially, H1299ρ^0^ cocultured with iCAFs-CM possessed these mutant sites as well (Fig. 5E), indicating that mtDNA from iCAFs could be transmitted to H1299ρ^0^ cells. Furthermore, mito-Red was used to label mitochondria to measure its function. Compared with H1299ρ^0^/A549ρ^0^, H1299/A549 had higher mitochondrial activity. And H1299ρ^0^/A549ρ^0^ cocultured with iCAFs-CM also possessed more mitochondrial activity than H1299ρ^0^/A549ρ^0^ treated with NFs-CM (Fig. 5F). In addition, H1299ρ^0^/A549ρ^0^ cocultured with iCAFs-CM also possessed more MMP levels than H1299ρ^0^/A549ρ^0^ treated with NFs-CM (Fig. 5G). Taken together, these data demonstrated the recovery of mtDNA in mitochondria damaged cells was from iCAFs-mediated mtDNA transfer but not an induction of mtDNA endogenous expression.

#### Mitochondria damaged tumor cells restores mitochondrial function, the ability of cell proliferation, migration and EMT through uptaking mtDNA released by iCAFs

**Fig. 6:**
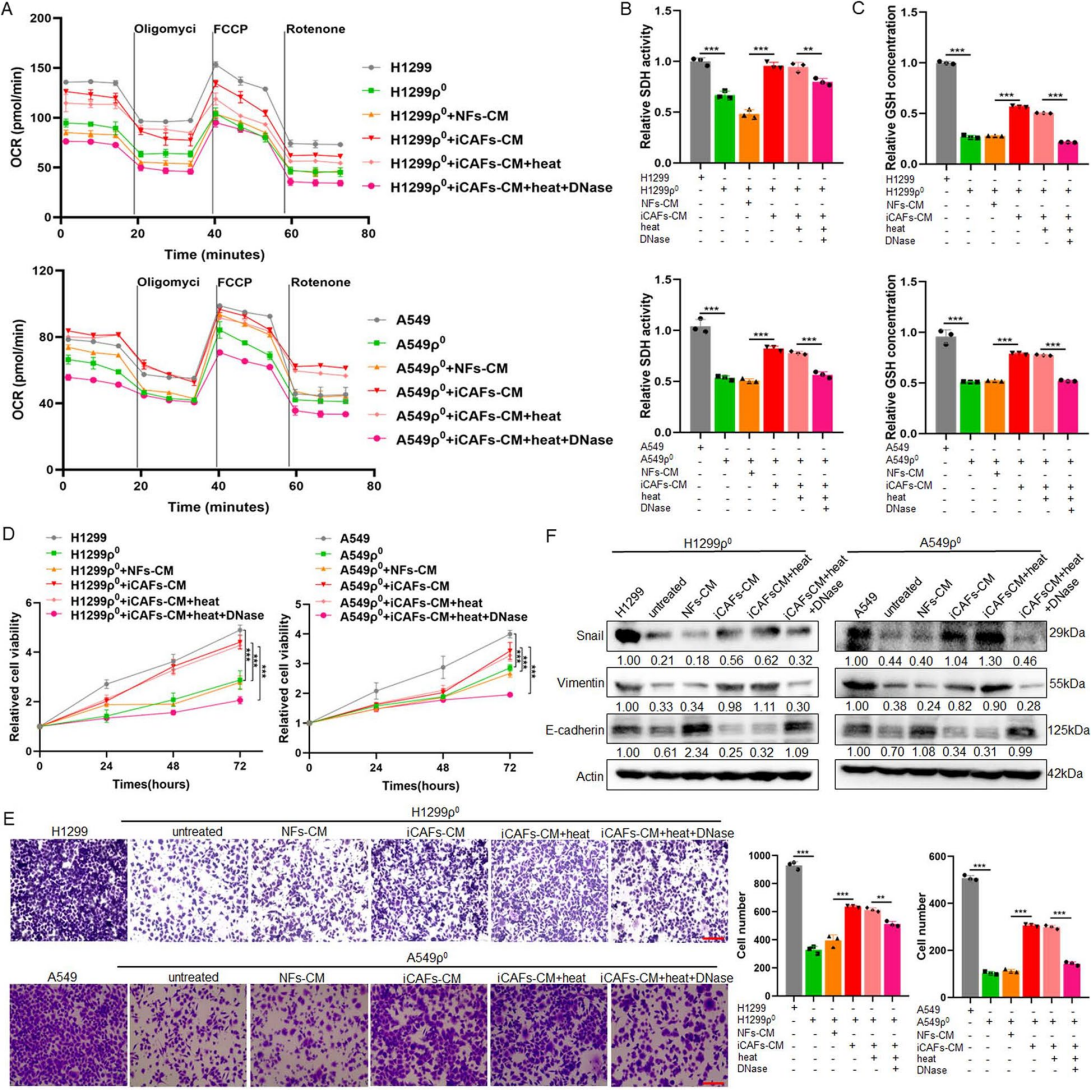
Mitochondria damaged tumor cells restores mitochondrial function, the ability of cell proliferation, migration and EMT through uptaking mtDNA released by iCAFs. Used H1299, A549, H1299ρ^0^ and A549ρ^0^ cells as control groups, cocultured H1299ρ^0^ and A549ρ^0^ cells with NFs-CM or iCAFs-CM for 48 h, respectively, and the CM was treated with DNaseI enzyme (0.1 mg/mL) in 37 °C for 1 h to hydrolyze mtDNA and followed by heating (70 °C for 10 min) to inactivate enzyme. **A** OCR was detected by seahorse mitochondrial stress. **B, C** The activity of SDH and the concentration of GSH were detected. **D** CCK8 assay for cell proliferation. **E** Transwell assays for the cell migration analysis. Scale bar, 100 μm. **F** Immunoblotting analysis of E-cadherin, Vimentin and Snail expression. Data were shown as the mean ± SD of at least three independent experiments. **P < 0.01, ***P < 0.001

To investigate the effect of mtDNA from CAFs on mitochondria damaged tumor cells, after H1299ρ^0^/A549ρ^0^ cells coculture with NFs-CM or iCAFs-CM, meanwhile CM was treated with DNaseI enzyme to hydrolyze mtDNA and followed by heating to inactivate enzyme [28], the OCR, SDH activity and GSH concentration were examined. The data found that H1299ρ^0^/A549ρ^0^ cells coculture with iCAFs-CM could restore the mitochondrial functions. Heating treatment does not affect this effect, whereas the addition of DNaseI into iCAFs-CM to degrade the DNA resulted in impaired mitochondrial function recovery (Fig. 6A, B, C). Further, CCK8 and transwell assays results revealed that iCAFs-CM significantly enhanced cell proliferation and migration compare with NFs-CM, and the addition of DNaseI could weaken this ability (Fig. 6D, E). In addition, the immunoblotting results showed that compared to control groups, the epithelial-mesenchymal transition (EMT) marker Vimentin and Snail were upregulated in H1299ρ^0^/A549ρ^0^ cocultured with iCAFs-CM, while the expression of epithelial marker E-cadherin was downregulated. However, under DNaseI treatment, this regulation was blocked (Fig. 6F, Fig. S7). These results suggest that mtDNA from CAFs can be taken up by tumor cell depleted of mtDNA, thus contributing to its mitochondrial function, the ability of cell proliferation and migration and EMT recovery.

#### iCAFs promote the growth and metastasis of mitochondria damaged tumor cells in vivo

**Fig. 7:**
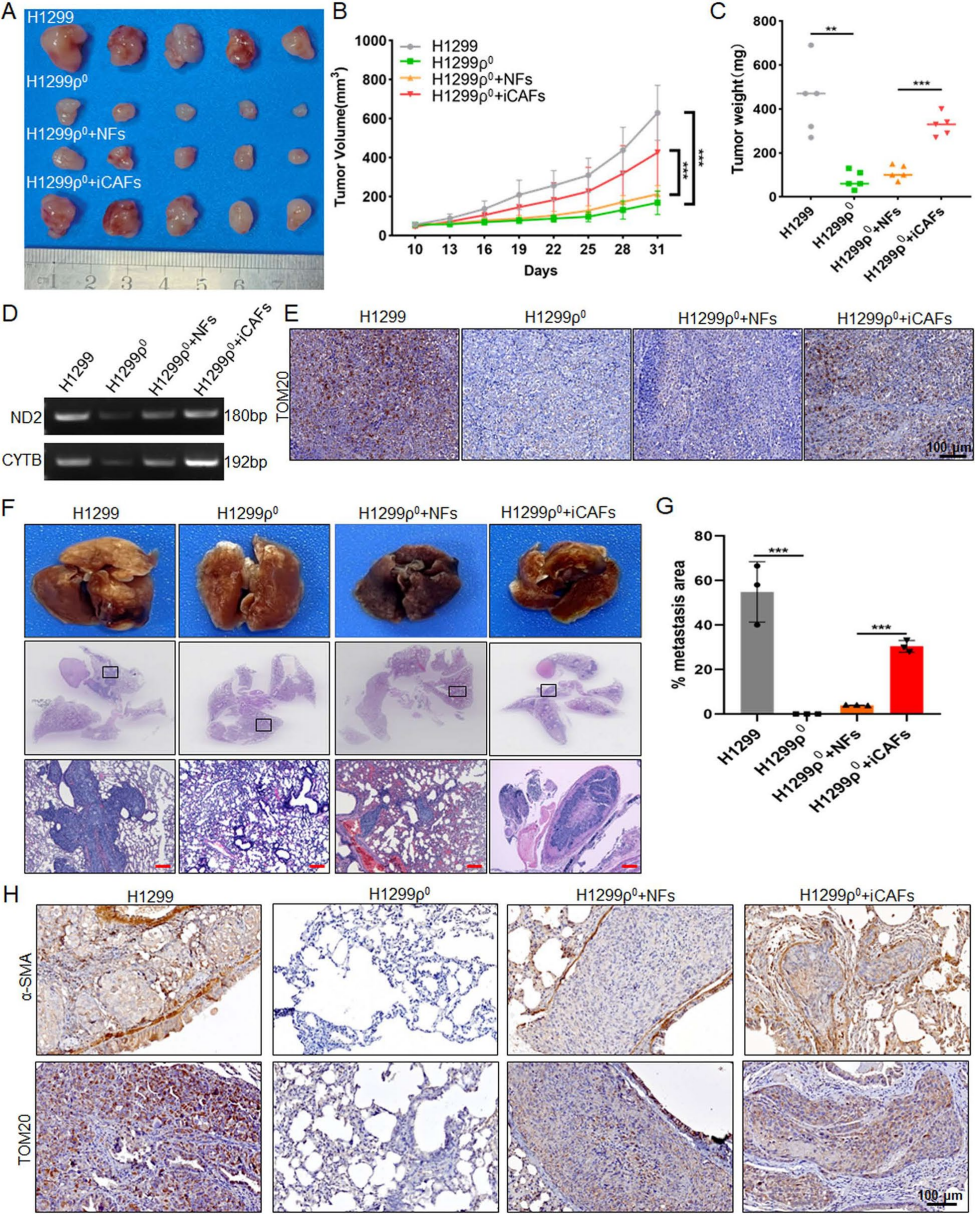
iCAFs promote the growth and metastasis of mitochondria damaged tumor cells in vivo. **A-C** After H1299 (2 × 10^6^), H1299ρ^0^ cells (2 × 10^6^), the mixture of 2 × 10^6^ H1299ρ^0^ cells with 5 × 10^5^ NFs or 5 × 10^5^ CAFs cells (4:1) were injected subcutaneously into 5-week-old nude mice, **A** the tumor growth, **B** tumor volume and **C** tumor weight was monitored. **D** PCR was used to detect the ND2 and CYTB in the indicated groups. **E** IHC analysis of TOM20 was performed in mouse xenograft tissues. **F–H** After H1299 (5 × 10^5^), H1299ρ^0^ cells (5 × 10^5^), the mixture of 5 × 10^5^ H1299ρ^0^ cells with 5 × 10^5^ NFs or 5 × 10^5^ iCAFs cells (1:1) were injected into the lateral tail vein of 5-week-old nude mice, **F** the representative tissue macrographs and HE images of lung foci, and **G** quantitative analysis of lung metastatic foci. Scale bar, 200 μm. **H** IHC images of α-SMA and TOM20 in lung foci of different experimental groups. Data were shown as the mean ± SD of at least three independent experiments. **P < 0.01, ***P < 0.001

To confirm the iCAFs could promote the growth and metastasis of mitochondria damaged tumor cells in vivo, subcutaneous xenograft experiment was performed by using H1299, H1299ρ^0^ and H1299ρ^0^ co-injected with NFs/iCAFs cells in the axilla of nude mice. And the results showed that the tumorigenic ability of H1299ρ^0^ was significantly weaker than that of H1299 cells, while co-injection with NFs slightly increased the tumorigenic capacity of H1299ρ^0^, but it is significantly weaker than the group co-injected with iCAFs (Fig. 7A, B). The same conclusion was obtained in the tumor weight results (Fig. 7C). TOM20 is widely used to monitor the mitochondrial mass, and a decreased expression of TOM20 is considered to indicate a reduced mitochondrial mass and upregulated mitophagy [29]. To demonstrate the transfer of mtDNA and the restoration of mitochondrial function in mitochondria damaged tumor cells in vivo, the PCR and IHC results showed that the group H1299ρ^0^ co-injected with iCAFs restores the mtDNA expression and exhibits higher level of mitochondria marker TOM20 compared with co-injection with NFs, which is closely related to their metastatic mtDNA and the restoration of tumor cell function (Fig. 7D, E). Moreover, H1299, H1299ρ^0^ cells and the mixture of H1299ρ^0^ cells with NFs or iCAFs cells were injected nude mice via the tail vein to conduct the lung cancer metastasis formation model, respectively. The results demonstrated that the metastatic foci in the lungs of the group co-injected with H1299ρ^0^ and iCAFs were more significant than those in the group co-injected with NFs (Fig. 7F,G). To determine the effect of iCAFs in lung metastatic foci, the IHC results showed that the expression of α-SMA and TOM20 was significantly higher in mice engrafted with H1299ρ^0^ treated with iCAFs compared with NFs (Fig. 7H). Together, these results suggest that iCAFs could promote the growth and metastasis of mitochondria damaged tumor cells in vivo.

## Discussion

It has been found that tumor cells undergoing oxidative stress can lead to mitochondrial dysfunction, which is closely associated with reduce of mtDNA copy number [8, 9]. Since Spees et al. has proved that mitochondria or mtDNA can move between cells [30], studies further point out that mitochondria or mtDNA could be transported between stromal cell and cancer cells, thereby restoring mitochondrial function in mitochondria damaged tumor cells and promoting tumor progress. For example, the study showed a preferential transfer of mitochondria from endothelial to cancer cells resulting in acquisition of chemoresistance [31]. Mitochondria damaged cancer cells that acquire mtDNA from host stroma restore their mitochondria function, thereby exerting a pro-cancer function [19]. And the mtDNA can be transferred via EVs from stromal cells to cancer cells to sustain OXPHOS potential and mediate an exit from hormonal therapy-induced metabolic dormancy in breast cancer [32]. In present study, our data showed that tumor cells with compromised respiration due to mtDNA depletion have the following characteristics: decreased oxygen consumption, downregulated levels of oxidative stress related enzymes and increased levels of ROS. Specially, the mitochondrial genome sequencing data show that the mtDNA mutation in cancer cell was different from CAFs, the mtDNA SNP sequencing results further support that mtDNA could be the paracrine-signaling molecule generated by CAFs and could be absorbed by cancer cell with mtDNA deletion. And the rescue of mitochondrial function in cancer cell with mtDNA deletion cocultured with CAFs-CM was demonstrated.

CAFs are one of the most prominent and active components in the lung cancer microenvironment, and the enhancement of autophagy in CAFs has been shown to play a role in the malignant phenotype of human tumors. Autophagy promotes tumor progress not only by providing nutrients to the cancerous cells but also by inducing EMT, angiogenesis, stemness, and metastatic dissemination of the cancer cells [33]. Yuan et al. demonstrated that the nucleosides are secreted by CAFs through autophagy increased glucose utilization and promoted growth of pancreatic ductal adenocarcinoma [34]. Li et al. report shows that rocuronium bromide could repress PI3K/AKT/mTOR signaling pathway and autophagy to block the CXCL12 expression in CAFs, thereby weakening the cytokines CXCL12-mediated esophageal cancer progression [35]. A well-studied mechanism that triggers the reverse warburg effect is ROS-induced mitophagy in CAFs. Martinez et al. showed that cancer cells triggered oxidative stress-induced mitophagy in neighboring fibroblasts by secreting hydrogen peroxide, thereby facilitating stromal aerobic glycolysis [36]. ITGB4-overexpressing triple negative breast cancer cells provided CAFs with ITGB4 proteins via exosomes, which induced BNIP3L-dependent mitophagy and lactate production, and then promoted the proliferation, EMT and invasion of breast cancer cells [37]. Our results showed that iCAFs exhibited a higher level of autophagy and mitophagy, and ROS could further enhance mitophagy and increase release of mtDNA in CAFs. This further prove the communication between CAFs and tumor cells through autophagy and ROS.

Numerous studies have shown that tumor derived EVs play important roles in various events of tumor development including the activation of NFs, through the proteins, DNA and RNA substances they carry. The molecules that induce CAF activation vary in different tumor cells [38]. The exosomal miR-210 derived from tumor cells induces the reprogramming of NFs to CAFs through TET, further promotes tumor angiogenesis and progress by producing MMP9, FGF2 and VEGF [39]. Lung cancer cell derived-EVs packaged lncRNA HOTIAR could motivate the transformation of NFs into CAFs, thereby promote the tumor metastasis [40]. In our study, we analyzed the different profiles of miRNAs carried by EVs between high-metastatic cancer cells and low-metastatic lung cancer cells, and identified that miR-1290 expression was higher both in high-metastatic cancer cells and EVs. Previous studies have shown that serum exosomal miR-1290 could be a potential diagnostic and prognostic biomarker for lung adenocarcinoma [41], and is a crucial driver for tumor initiation and cancer progression in human non-small cell lung cancer [22]. A study showed that hypoxic lung adenocarcinoma cell-derived exosomes overexpressing miR-1290 can polarize M2 macrophages by targeting SOCS3, which further promote tumor progression [42, 43]. And recent report has found that exosomal miR-1290 improves CAFs activation through target COX-2, further enhance tumor growth [42, 43]. Here, we found that miR-1290 directly transferred from high-metastatic cancer cells to NFs via EVs, and further activate NFs to CAFs through MT1G/AKT pathway. However, it should be noted that miR-1290 is not the only cargos packaged in EVs, other components also have a certain effect, for example, 95C-EVs with low miR-1290 expression could activate NFs to a certain extent, these still need further clarification.

## Conclusions

**Fig. 8:**
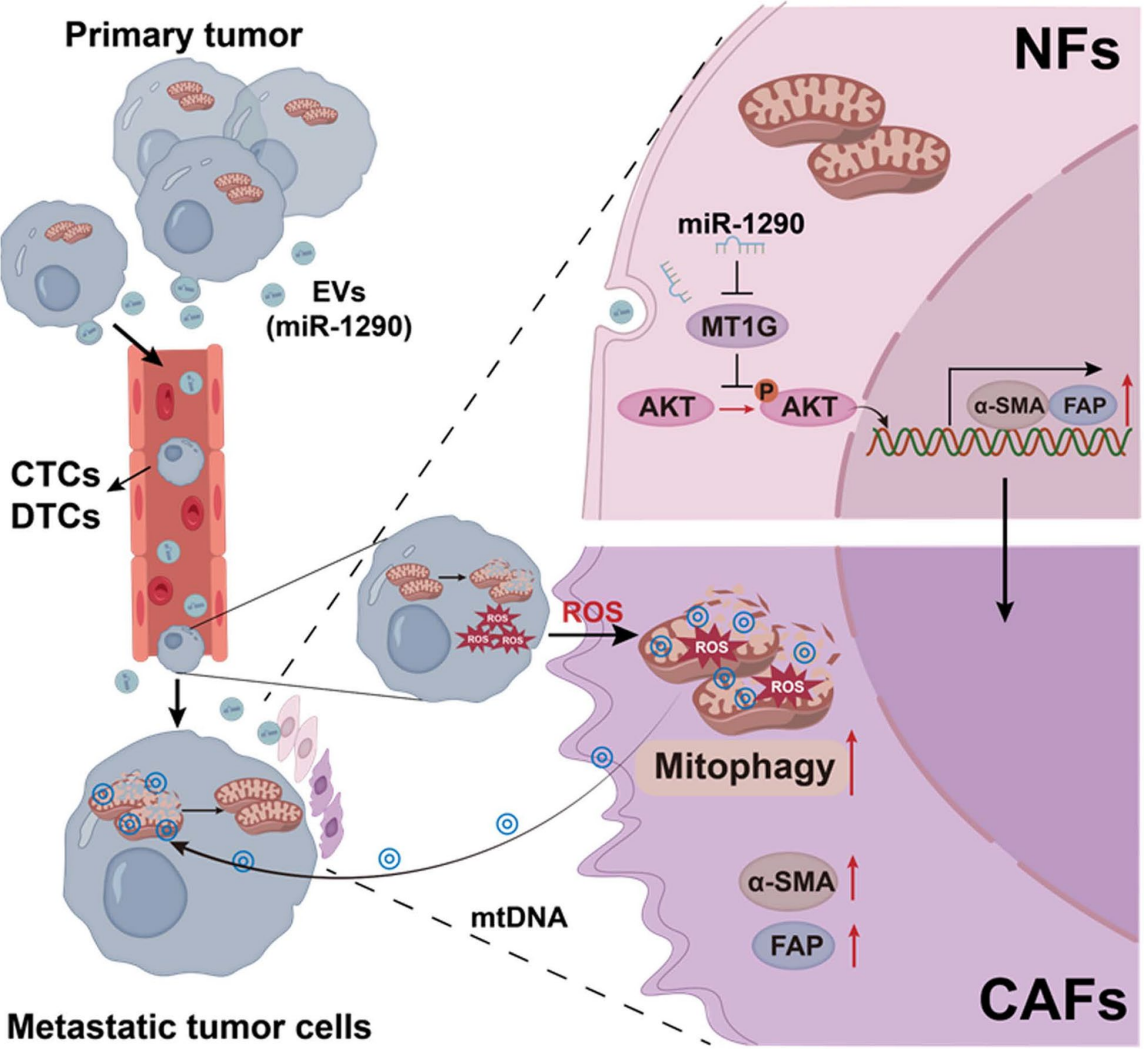
Schematic diagram**.** The role and mechanism of EVs activated CAFs in regulating lung cancer metastasis through mitophagy and mtDNA transfer

In summary, our results indicate that high metastatic lung cancer cells derived EVs packaged miR-1290 promote the activation of NFs to CAFs. The activated CAFs exhibit higher levels of autophagy and mitophagy and more mtDNA release, and ROS could further promote this process. More importantly, tumor cells with compromised mitochondrial due to mtDNA depletion, could absorb the mtDNA provided by CAFs, thus promote the recovery of mitochondrial function and resistance to oxidative stress, thereby promote tumor metastasis (Fig. 8). Our study elucidates a novel mechanism by which intercellular crosstalk between tumor cells and CAFs control lung cancer metastasis, providing potential efficient prevention and therapeutic strategies for lung cancer.

### Supplementary Information

Supplementary Material 1.

## Data Availability

All the data supporting the findings of this study are available within the article and its additional files.

## Abbreviations

mtDNA: Mitochondrial DNA
NFs: Normal fibroblasts
CAFs: Cancer-associated fibroblasts
TME: Tumor microenvironment
EVs: Extracellular vesicles
iCAFs: Induced CAFs
ROS: Reactive oxygen species
CM: Conditioned medium
ECM: Extracellular matrix
CTCs: Circulating tumor cells
DTCs: Disseminated tumor cells
FBS: Fetal bovine serum
EtBr: Ethidium bromide
H_2_O_2_: Hydrogen peroxide
CCCP: Carbonyl cyanide 3-chlorophenylhydrazone
TEM: Transmission electron microscopy
NTA: Nanoparticle tracking analysis
IF: Immunofluorescence
qPCR: Quantitative PCR
MMP: Mitochondrial membrane potential
SDH: Succinate dehydrogenase
GSH: Glutathione
OCR: Oxygen consumption rate
SNP: Single nucleotide polymorphism
ISH: In situ hybridization
IHC: Immunohistochemistry
MT1G: Metallothionein 1G
EMT: Epithelial-mesenchymal transition
