# Supplementary material for: Extracellular vesicles activated cancer-associated fibroblasts promote lung cancer metastasis through mitophagy and mtDNA transfer
Source: J Exp Clin Cancer Res. 2024 Jun 3;43:158. doi: 10.1186/s13046-024-03077-w (PMC11145873; doi:10.1186/s13046-024-03077-w)
Supplement: Supplementary file 1 — Supplementary Material 1. [file 13046_2024_3077_MOESM1_ESM.docx]

**Extracellular vesicles** **activated** **cancer-associated fibroblasts promote lung cancer metastasis through mitophagy and mtDNA transfer**

Zhuan Zhou, Chunhui Qu, Peijun Zhou, Qin Zhou, Dan Li, Xia Wu, Lifang Yang

**Supplemental Figures and Tables**

**Supplemental Figures**

**
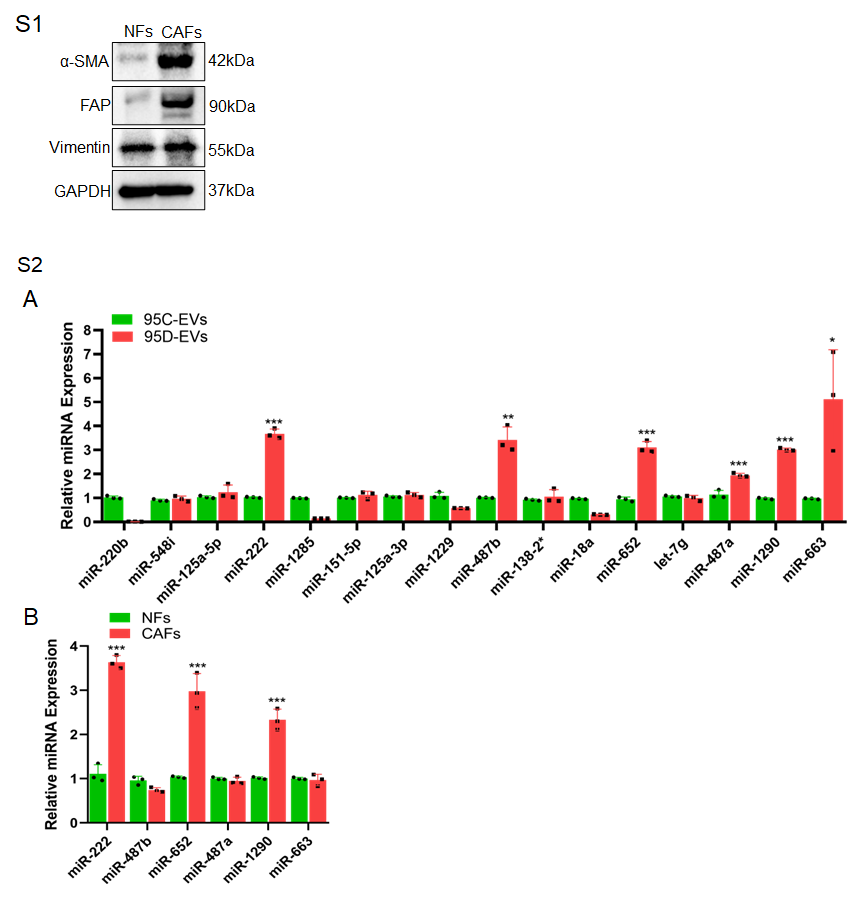
**

**Supplementary Fig.1.** **NFs and CAFs cells were identified.** Immunoblotting analyses for the [mesenchyme](https://www.sciencedirect.com/topics/medicine-and-dentistry/mesenchyme) marker protein Vimentin and CAF marker protein α-SMA and FAP in NFs and CAFs.


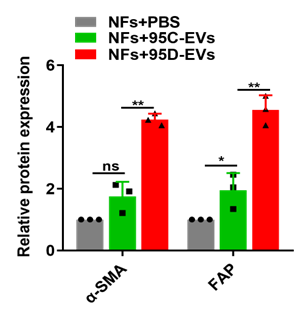


**Supplementary Fig.2.** **95D-derived EVs activated NFs to CAFs.** After NFs were incubated with 95C or 95D derived EVs for 48 h, the protein levels of α-SMA and FAP were analyzed by immunoblotting. Data were shown as the mean ± SD of three independent experiments. *P < 0.05, **P < 0.01, ns: not significant.


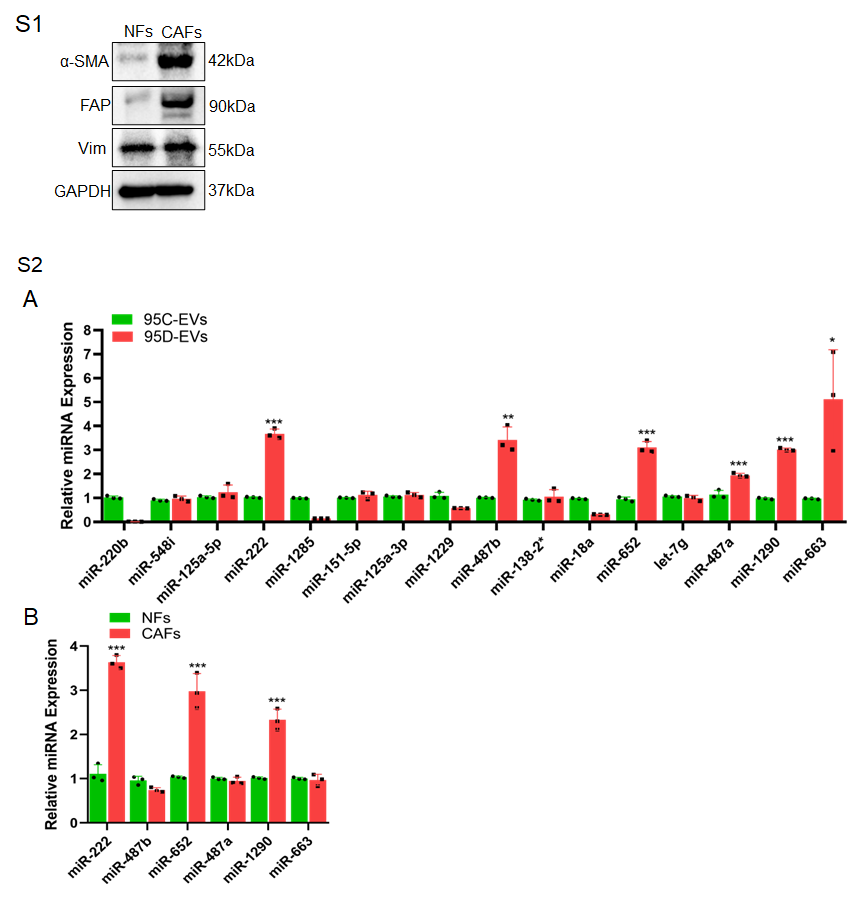


**Supplementary Fig.3.** **miR-1290, miR-222 and miR-652 were highly expressed in 95D-derived EVs and CAFs. (A)** qPCR was used to detect differentially expressed miRNAs in EVs derived from 95D cells compared with 95C cells. **(B)** miRNAs were determined by qPCR in CAFs compared with NFs. Data were shown as the mean ± SD of at least three independent experiments. *P < 0.05, **P < 0.01, ***P < 0.001.


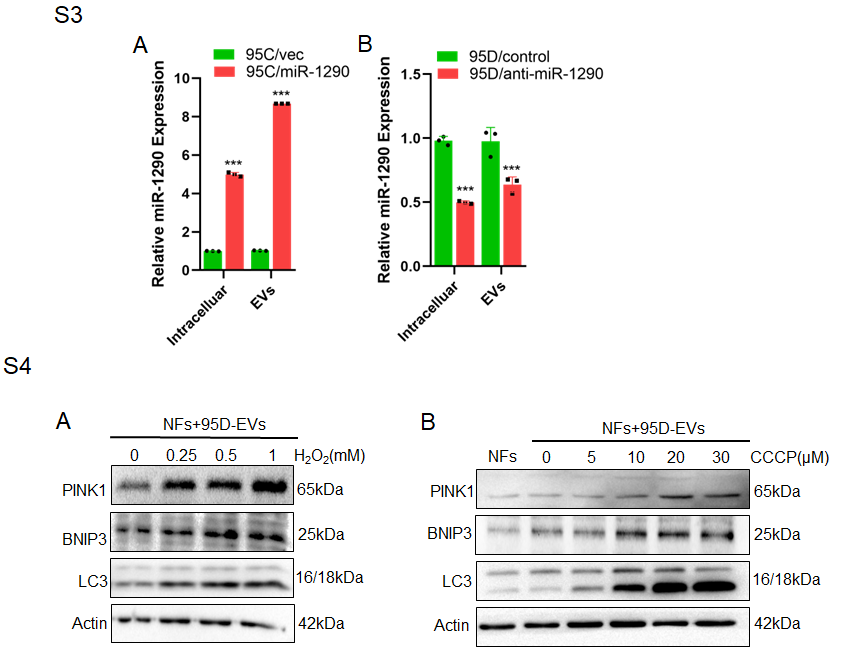


**Supplementary Fig.4.** **The expression of miR-1290 in stable cell lines 95C/miR-1290 and 95D/anti miR-1290.** qPCR was used to detect miR-1290 in intracell and EVs derived from **(A)** 95C/vec and 95C/miR-1290, **(B)** 95D/control and 95D/anti-miR-1290. Data were shown as the mean ± SD of at least three independent experiments. ***P < 0.001.


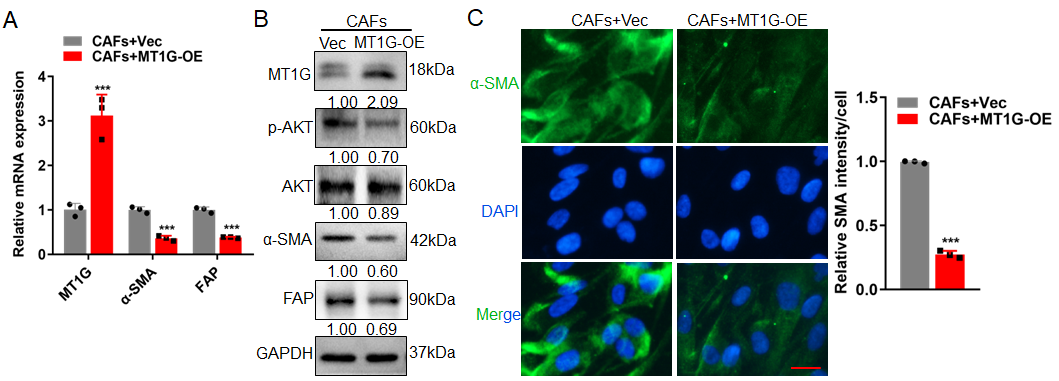


**Supplementary Fig.5.** **Overexpression of MT1G inhibited AKT-mediated activation of CAFs.** (A-C) After CAFs transfected with pDONR233-MT1G plasmid or empty plasmid (Vec), (A) qPCR assays for MT1G, α-SMA and FAP, and (B) Immunoblotting analysis of MT1G, p-AKT, AKT, α-SMA and FAP were performed. (H) IF analysis and quantification data of α-SMA. Scale bar, 20 μm. Data were shown as the mean ± SD of at least three independent experiments. ***P < 0.001.


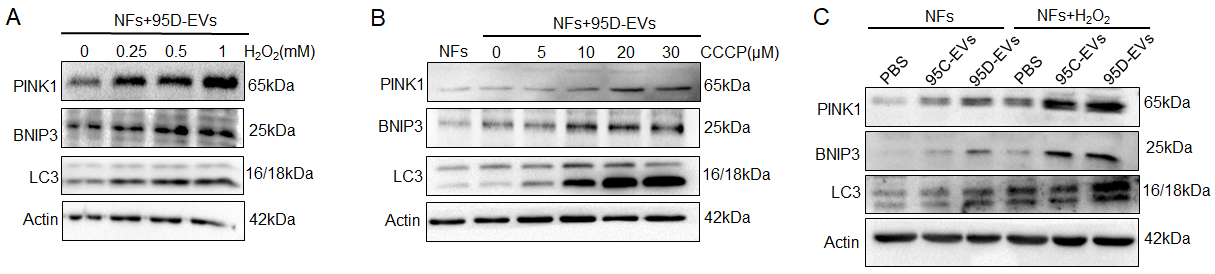


**Supplementary Fig.6. H_2_O_2_ and CCCP induced mitochondrial autophagy.** **(A)** Immunoblotting analyses for protein levels of PINK1, BNIP3 and LC3 in NFs cocultured with 95D-EVs followed by treating with H_2_O_2_ (0, 0.25, 0.5, and 1 mM) for 12 h. **(B)** Immunoblotting analyses for protein levels of PINK1, BNIP3 and LC3 in NFs cocultured with 95D-EVs followed by treating with CCCP (0, 5, 10, 20 and 30 μM) for 24 h. **(C)** NFs were treated with EVs derived from 95C or 95D for 48 h, and followed by treating H_2_O_2_ (0.25 mM) for 12 h. Immunoblotting analysis of PINK1, BNIP3 and LC3 in the indicated groups.

**
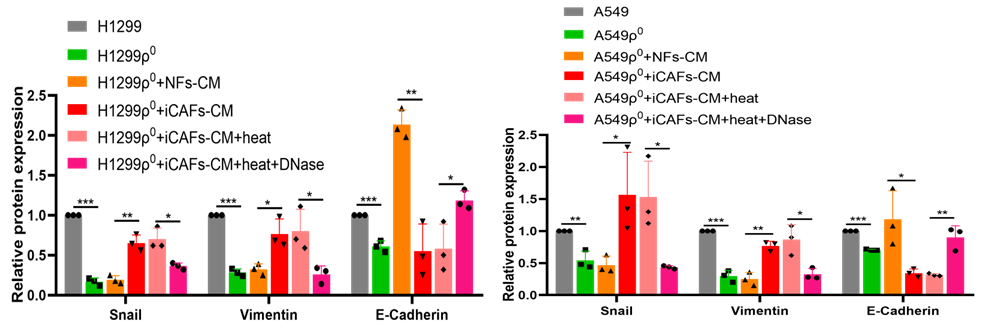
**

**Supplementary Fig.7.** **Mitochondria damaged tumor cells restores EMT through uptaking mtDNA released by iCAFs.** Used H1299, A549, H1299ρ^0^ and A549ρ^0^ cells as control groups, cocultured H1299ρ^0^ and A549ρ^0^ cells with NFs-CM or iCAFs-CM for 48 h, respectively, and the CM was treated with DNaseⅠ enzyme (0.1 mg/mL) in 37 °C for 1 h to hydrolyze mtDNA and followed by heating (70°C for 10 min）to inactivate enzyme. Statistical analysis of immunoblotting detected of Snail, Vimentin and E-cadherin protein expression. Data were shown as the mean ± SD of three independent experiments. *P < 0.05, **P < 0.01, ***P < 0.001.

**Supplemental Figures and Tables**

**Table S1. Primers for PCR**

| **Gene** | **Forward Primer** | **Reverse Primer** |
| --- | --- | --- |
| D-LOOP | GTCTATCACCCTATTAACCACTC | CGTCTCGCAATGCTATCG |
| 16S | AACTTTGCAAGGAGAGCCAAAGC | ACGCCATAAAACTCTTCACCAAAG |
| ATP6 | CGCCTAACCGCTAACATTAC | TTGTGAAGATGATAAGTGTAGAGG |
| ATP8 | ATGGCCCACCATAATTACCC | CATTTTGGTTCTCAGGGTTTG |
| COX1 | TGCCATAACCCAATACCAAACGC | CTGTTAGTAGTATAGTGATGCCAGCAGCTAGG |
| COX2 | CTACGGTCAATGCTCTGAAATCTGTG | GCTAAGTTAGCTTTACAGTGGGCTCTAG |
| COX3 | CGATACGGGATAATCCTATTTATTACCTCAG | CAGGTGATTGATACTCCTGATGCGA |
| ND3 | CATTTTGACTACCACAACTCAACGGCTAC | GGGTAAAAGGAGGGCAATTTCTAGATC |
| ND4 | GCTACTCTCATAACCCTCAACACCC | AGGCCATATGTGTTGGAGATTGAGA |
| ND4 | CCAACGCCACTTATCCAGTG | GGGAAGGGAGCCTACTAGGGTGT |
| ND5 | TTACCACCCTCGTTAACCCTAACAAA | TGGGTTGTTTGGGTTGTGGCT |
| ND6 | ACGCCCATAATCATACAAAGCCC | GGATTGGTGCTGTGGGTGAAA |
| CYTB | CGCCTGCCTGATCCTCCAA | AGGCCTCGCCCGATGTGTAG |
| ND2 | TCTTATCCATCATAGCAGGCAGTTG | GTAGTAGGAATGCGGTAGTAGTTAGG |
| 12S | ACCTCACCACCTCTTGCTC | GGCTACACCTTGACCTAACG |
| ND1 | GCAGAGACCAACCGAACC | TATGAAGAATAGGGCGAAGGG |
| FAP | TGGTATAGCAGTGGCTCCAGTCTC | ATCTGCTGTTCCGTGGATGAGAAG |
| ACTA2 | CTCTGGACGCACAACTGGCATC | CACGCTCAGCAGTAGTAACGAAGG |
| MT1G | AGAGTGCAAATGCACCTCCTGC | TTGTACTTGGGAGCAGGGCTGT |
| ACTIN | CATGTACGTTGCTATCCAGGC | CTCCTTAATGTCACGCACGAT |

**Table S2. Primers for miRNA PCR**

| **miRNA** | **Primer** |
| --- | --- |
| has-miR-1290 | CGGTGGATTTTTGGATCAGGGA |
| has-miR-138-2 | GCTATTTCACGACACCAGGGTT |
| hsa-miR-487a | CGGAATCATACAGGGACATCCAGTT |
| hsa-miR-151-5p | TCGAGGAGCTCACAGTCTAGT |
| hsa-miR-125a-3p | ACAGGTGAGGTTCTTGGGAG |
| hsa-miR-220b | CCACCACCGTGTCTGACACTT |
| hsa-miR-1229 | TAATACTCTCACCACTGCCCTCC |
| hsa-miR-125a-5p | TCCCTGAGACCCTTTAACCTGTGA |
| hsa-miR-18a | GCTAAGGTGCATCTAGTGCAGATAG |
| hsa-miR-487b | CGAATCGTACAGGGTCATCCACTT |
| hsa-miR-652 | AATGGCGCCACTAGGGTTGTG |
| hsa-miR-1285 | TCTGGGCAACAAAGTGAGACCT |
| hsa-miR-663 | AGGCGGGGCGCCGCGGGACCGC |
| hsa-let-7g | GCGTGAGGTAGTAGTTTGTACAGTT |
| hsa-miR-222-3p | AGCTACATCTGGCTACTGGGT |
| hsa-miR-548i | CGGAAAAGTATTTGCGGGTTTTGTC |

**Table S3. Clinical characteristics of 41 lung cancer patients**

| **Characteristics** | **Adenocarcinoma**  Number of patients (%) | **Squamous cell carcinoma**  Number of patients (%) |
| --- | --- | --- |
| **Gende**r |  |  |
| Male | 13(59.09%) | 17(89.47%) |
| Female | 9(40.91%) | 2(10.53%) |
| **Age** |  |  |
| ≥50 | 17(59.09%) | 18(89.47%) |
| <50 | 5(40.91%) | 1(10.53%) |
| **Clinic Stage** |  |  |
| Ⅰ | 18(81.82%) | 16(84.21%) |
| Ⅱ | 2(9.09%) | 0(0) |
| Ⅲ | 2(9.09%) | 3(15.79%) |
| **Lymph Nodes stage** |  |  |
| N0 | 19(86.36%) | 16(84.21%) |
| NⅠ | 1(4.55%) | 0(0) |
| NⅡ | 2(9.09%) | 3(15.79%) |
